# Analysis of Pharmacokinetic and Pharmacodynamic Interactions Between Chlorpromazine and Risperidone via Simultaneous Measurement of Multiple Receptor Occupancy in the Rat Brain

**DOI:** 10.3390/biomedicines14010118

**Published:** 2026-01-06

**Authors:** Gaku Akashita, Eriko Nakatani, Shimako Tanaka, Takashi Okura

**Affiliations:** Laboratory of Pharmaceutics, Faculty of Pharmaceutical Sciences, Teikyo University, Tokyo 173-8605, Japan; akashita-gaku@staff.kanazawa-u.ac.jp (G.A.); erikon@pharm.teikyo-u.ac.jp (E.N.); tanaka.shimako.kg@teikyo-u.ac.jp (S.T.)

**Keywords:** receptor occupancy, drug-drug interaction, dopamine 2 receptor, serotonin 2A receptor, histamine 1 receptor, muscarinic acetylcholine receptor, liquid chromatography, mass spectrometry, antipsychotics

## Abstract

**Background/Objectives:** Combination therapy for schizophrenia may exacerbate side effects mediated by multiple brain receptors. This study aimed to elucidate the pharmacodynamic and pharmacokinetic interactions between chlorpromazine and risperidone. We investigated dopamine 2 (D_2_), serotonin 2A (5-HT_2A_), histamine 1 (H_1_), and muscarinic acetylcholine (mACh) receptor occupancy in the brain as well as pharmacokinetic interactions after oral administration of chlorpromazine and risperidone in rats. **Methods:** Rats were orally administered chlorpromazine, risperidone, or their combination. A tracer cocktail solution was injected intravenously to measure multiple receptor occupancies simultaneously. Tracer and drug concentrations in the brain tissue and plasma were quantified by liquid chromatography-tandem mass spectrometry (LC-MS/MS). **Results:** Receptor occupancy increased in a dose-dependent manner. The doses required for 70% D_2_ receptor occupancy were 4.5 mg/kg for chlorpromazine and 1.5 mg/kg for risperidone. Co-administration of chlorpromazine (4.5 mg/kg) and risperidone (1.5 mg/kg) resulted in an increase in D_2_ and 5-HT_2A_ receptor occupancy to approximately 90%. Risperidone alone caused a transient increase in H_1_ receptor occupancy to 80%, while co-administration increased mACh receptor occupancy to 60%. Co-administration with chlorpromazine significantly increased the plasma concentrations of risperidone and its metabolite, paliperidone, and decreased the oral clearance of risperidone by 5.9-fold. **Conclusions:** Co-administration of chlorpromazine and risperidone increases the occupancy of D_2_, 5-HT_2A_, and mACh receptors in the rat brain and increases the plasma concentrations of risperidone and paliperidone, suggesting a potential risk of enhanced adverse effects due to both pharmacokinetic and pharmacodynamic interactions involving target and non-target brain receptors.

## 1. Introduction

Schizophrenia is a severe mental illness characterized by hallucinations, delusions, disorganized thinking and speech, and impaired social functioning. Its symptoms are classified as positive and negative [1]. First-generation antipsychotics, which primarily block dopamine 2 (D_2_) receptors, are used to treat the positive symptoms, whereas second-generation antipsychotics, which block serotonin 2A (5-HT_2A_) and D_2_ receptors, are used to treat the negative symptoms [2,3,4]. Depending on the symptoms of schizophrenia, monotherapy or combination therapy with first-generation and second-generation antipsychotics such as chlorpromazine and risperidone, respectively, are used. The rate of combination therapy is reported as 2.6–95.2% [5]. Combination therapy with first- and second-generation antipsychotics increases D_2_ receptor occupancy, and when occupancy exceeds the therapeutic range (65–80%), the risk of extrapyramidal symptoms increases [6,7,8,9,10]. These clinical thresholds closely parallel those observed in rat studies, where suppression of the conditioned avoidance response occurs at 70–75% D_2_ receptor occupancy, and catalepsy manifests above 80% [11]. Chlorpromazine-based equivalent values are used to avoid any pharmacodynamic interactions. In a face-to-face survey of 48 patients in Japan receiving antipsychotic therapy, 98% reported experiencing side effects, including anticholinergic symptoms (68%) and extrapyramidal disorders (77%) [12].

In combination therapy, pharmacokinetic and pharmacodynamic interactions should be considered among the pharmacological agents. Many antipsychotics, including chlorpromazine and risperidone, are substrates of cytochrome P450 2D6 enzyme (CYP2D6) [13], and metabolic inhibition can increase their plasma concentrations, increasing the risk of side effects. Furthermore, many antipsychotics, such as chlorpromazine and risperidone, have been reported to exhibit affinity for non-target receptors, such as histamine 1 (H_1_) and muscarinic acetylcholine (mACh) receptors, in addition to their target receptors D_2_ and 5-HT_2A_ [14,15]. A central H_1_ receptor occupancy of at least 50% has been shown to produce sedative effects [16], and the blockade of mACh receptors poses a risk of cognitive decline [17,18]. Therefore, combination therapy may potentially exacerbate side effects mediated by multiple brain receptors, including histamine H_1_ and mACh receptors, in addition to dopamine D_2_ and 5-HT_2A_ receptors.

To analyze the occupancy of multiple brain receptors by antipsychotic drugs, we established a method that enables simultaneous assessment of the occupancy of multiple receptors, including the D_2_, H_1_ and mACh receptors, by administering a cocktail of unlabeled tracers and separately quantifying each tracer via liquid chromatography-tandem mass spectrometry (LC-MS/MS) [19]. This method may be useful for determining drug-drug interactions on the occupancy of multiple brain receptors during combination therapy with antipsychotic drugs. In this study, we examined pharmacodynamic and pharmacokinetic interactions between chlorpromazine and risperidone by assessing D_2_, 5-HT_2A_, H_1_, and mACh receptor occupancy and pharmacokinetic interactions in the rat brain after oral co-administration of chlorpromazine and risperidone.

## 2. Materials and Methods

### 2.1. Compounds

Raclopride and paliperidone were purchased from Tokyo Chemical Industry Co., Ltd. (Tokyo, Japan). MDL-100907 was purchased from Merck KGaA., Ltd. (Darmstadt, Germany). Pyrilamine maleate, propranolol hydrochloride, chlorpromazine hydrochloride, risperidone, and formic acid were purchased from FUJIFILM Wako Pure Chemical Corporation (Osaka, Japan). 3-Quinuclidinyl benzilate (3-QNB) was synthesized by Toronto Research Chemicals, Inc. (Toronto, ON, Canada).

### 2.2. Animals

All animal procedures were performed in accordance with the Teikyo University Animal Experiment Regulations and Ethics Committee Guidelines for Conducting Animal Experiments and were approved by the Teikyo University Animal Ethics Committee (19-040, 25 March 2020). Male Wistar rats (7-week-old, 130–190 g) were purchased from Japan SLC, Inc. (Hamamatsu, Japan). Three rats were housed per cage in a room under a 12-h light/dark period (lights on at 8:00 AM). The temperature was maintained at 24 ± 1 °C with a relative humidity of 55 ± 5%. Food and water were provided ad libitum.

### 2.3. Drug Administration and Blood and Brain Tissue Sample Collection

Raclopride, MDL-100907, pyrilamine, and 3-QNB were used as tracers for D_2_, 5-HT_2A_, H_1_ and mACh receptors, respectively. The dose-dependent experiments were conducted to estimate effective doses of chlorpromazine and risperidone at which striatal D_2_ receptor occupancy reaches 70% of the therapeutic range (65–80%) of antipsychotics including chlorpromazine and risperidone in the human and rat study [6,7,8,9,10,11]. Rats (*n* = 4 rats in each dose group) were orally administered vehicle, chlorpromazine (0.3, 1, 3, 10, and 30 mg/kg) or risperidone (0.01, 0.03, 0.1, 3, and 10 mg/kg), each dissolved in 25% 2-hydroxypropyl-β-cyclodextrin [20,21]. Thirty minutes after oral administration, a tracer cocktail solution (1 mL/kg) containing raclopride, MDL-100907, pyrilamine, and 3-QNB at doses of 3, 3, 10, and 30 µg/kg, respectively, was injected into the saphenous vein under sevoflurane inhalation anesthesia. The rats were sacrificed under anesthesia 45 min after the injection. Blood samples were collected from the descending aorta using a heparin-coated syringe. The brains were immediately removed, and brain tissues (striatum, cerebral cortex, and cerebellum) were dissected and weighed. Blood samples were centrifuged for 4 min at 3000 rpm and 4 °C (Centrifuge 5424R; Eppendorf, Hamburg, Germany).

During the time-course experiments, the rats (*n* = 4 rats in each time point of each treated group) were orally administered vehicle or chlorpromazine (4.5 mg/kg) and/or risperidone (1.5 mg/kg). The tracer cocktail solution was injected before 45 min sacrifice. Plasma and brain tissues were sampled 1, 2, 4, and 8 h after oral administration.

In the pharmacokinetic experiments, the rats (*n* = 5 rats in each treated group) were orally administered 4.5 mg/kg chlorpromazine and/or 1.5 mg/kg risperidone, and blood samples (120 µL each) were collected from the jugular vein using a heparin-coated syringe at 0, 0.5, 1, 1.5, 2, 3, 4, 6, 8, and 12 h (total of 10 time points). Brain tissue and plasma samples were stored at −80 °C until concentration measurements.

### 2.4. Determination of Tracer and Test Compound Concentration

Quantitative analysis of the tracer, chlorpromazine, risperidone, and paliperidone concentrations was performed using LC-MS/MS. Previously weighed brain tissue samples were placed in conical 2.0 mL polypropylene screw cap tubes, to which four volumes (*w*/*v*) of acetonitrile containing 0.1% formic acid and propranolol as an internal standard (final concentration = 25 nM) were added. The samples were then homogenized for 1 min at 3200 rpm using a bead crusher (µT-12; TAITEC Co., Koshigaya, Japan) and centrifuged for 10 min at 15,000× *g* (Centrifuge 5424R). The supernatant was diluted with an equal volume of 0.1% formic acid and injected into an LC-MS/MS system.

The analyses were performed in accordance with previous studies [19]. The details of the analysis conditions and the validation data are provided in Supplementary Materials. Multiple reaction monitoring was performed using electrospray ionization in positive ion mode. Each protonated analyte [M + H]^+^ was monitored with the following mass transitions: 346.99 → 112.0 *m*/*z* for raclopride, 374.13 → 356.20 *m*/*z* for MDL-100907, 286.15 → 121.00 *m*/*z* for pyrilamine, 338.09 → 128.00 *m*/*z* for 3-QNB, 318.97 → 58.10 *m*/*z* for chlorpromazine, 411.07 → 191.10 *m*/*z* for risperidone, 427.03 → 207.00 *m*/*z* for paliperidone, and 260.08 → 116.00 *m*/*z* for propranolol, respectively. The gradient program was as follows: 95% of solution A and 5% of solution B from the start of analysis till 0.2 min, which was then changed to 20% of solution A and 80% of solution B from 0.2 min to 3.2 min; then, from 3.2 to 4.0 min, it was isocratic at 20% of solution A and 80% of solution B. At 4.01 min, the mobile phase was reverted to 95% solution A and 5% solution B (as in the initial phase), and the total analysis time was 4.5 min. The autosampler temperature was maintained at 4 °C and the injection volume was set at 5 µL.

The calibration curve of the brain tissue was linear from 0.3 to 30 pmol/g tissue for raclopride and MDL-100907, 0.3 to 300 pmol/g tissue for pyrilamine and 3-QNB, 1 to 100 ng/g tissue for chlorpromazine, and 0.3 to 30 ng/g tissue for risperidone and paliperidone. The calibration curve of plasma was linear from 0.3 to 30 pmol/mL for raclopride, MDL-100907, pyrilamine and 3-QNB, 1 to 100 ng/mL for chlorpromazine, and 0.3 to 30 ng/mL for risperidone and paliperidone.

### 2.5. Calculation of Receptor Occupancy

Receptor occupancy was calculated from the binding potential (BP) or specific binding (SB) of each tracer using the following equations [19]:

(1) $$D_{2}\;and\;{5\text{-}HT}_{2A}\;receptor\;occupancy=\frac{{BP}_{vehicle}-{BP}_{chlorpromazine\;and/or\;risperidone}}{{BP}_{vehicle}}$$

(2) $$H_{1}\;and\;mAch\;receptor\;occupancy=\frac{{SB}_{vehicle}-{SB}_{chlorpromazine\;and/or\;risperidone}}{{SB}_{vehicle}}$$

Cerebellar concentrations of raclopride and MDL-100907 were estimated to have nonspecific distributions. The binding potentials of raclopride to D_2_ receptors in the striatum and MDL-100907 to 5-HT_2A_ receptors in the cerebral cortex were calculated from the tissue concentration of each tracer as follows:

(3) $$BP\;of\;Raclopride=\frac{{Raclopride}_{striatum}-{Raclopride}_{cerebellum}}{{Raclopride}_{cerebellum}}$$

(4) $$BP\;of\;MDL\text{-}100907=\frac{{MDL\text{-}100907}_{cerebral\;cortex}-{MDL\text{-}100907}_{cerebellum}}{{MDL\text{-}100907}_{cerebellum}}$$

The cerebral cortical concentrations of pyrilamine and 3-QNB in rats pre-administered with the displacer were used to estimate their nonspecific distribution, as tracer concentrations in the cerebellum could not reliably represent the nonspecific distribution for H_1_ and mAch receptor occupancy measurements [19]. Doxepin and atropine were used as displacers for H_1_ and mACh receptors, respectively [19]. Pre-administration of doxepin (100 mg/kg i.p., 30 min before tracer injection) or atropine (100 mg/kg i.p., 30 min before tracer injection) reduced the cerebral cortical concentration of pyrilamine or 3-QNB to 67% and 16%, respectively, compared with vehicle-treated rats. The SB of each tracer to H_1_ and mACh receptors in the cerebral cortex was calculated by subtracting the nonspecific distribution from total cerebral cortical concentration. To determine receptor occupancy, the SB of each tracer was normalized by dividing it by the corresponding plasma tracer concentration.

### 2.6. Statistical Analysis

Experimental values were expressed as the mean ± SD. Sample size calculations were not performed a priori. The assumption of normality was assessed using the Shapiro-Wilk test, followed by Bonferroni-Holm correction. Student’s *t*-test with Bonferroni-Holm correction was used to evaluate the significance of pharmacokinetic parameters between mono- and co-administration groups using GraphPad prism version 9.5.1 for MacOS (GraphPad Software, Boston, MA, USA). Post-hoc power was calculated using G*power version 3.1.9.6 for MAC OS [22]. Statistical significance was set at *p* < 0.05.

## 3. Results

### 3.1. Dose Dependence of D_2_, 5-HT_2A_, H_1_, and mACh Receptor Occupancy After Oral Administration of Chlorpromazine or Risperidone in Rats

**Figure 1:**
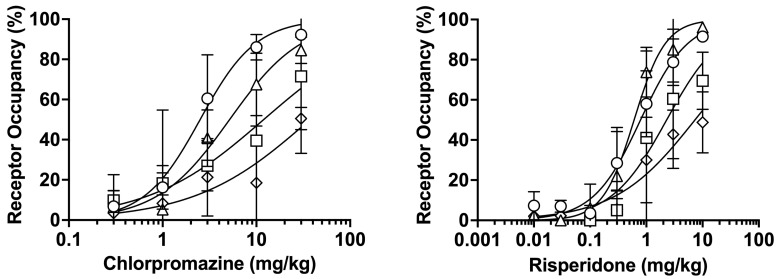
Dose-receptor occupancy curves for D_2_ (〇), 5-HT_2A_ (△), H_1_ (□), and mACh (◇) receptors after oral administration of chlorpromazine (0.3–30 mg/kg) or risperidone (0.01–10 mg/kg) for 75 min measured using raclopride, MDL-100907, pyrilamine, and 3-QNB as tracers. The data are presented as the mean ± standard deviation from four rats at each point.

Figure 1 shows the dose-receptor occupancy curves for D_2_, 5-HT_2A_, H_1_, and mACh receptors 75 min after oral administration of chlorpromazine or risperidone. Chlorpromazine and risperidone increased receptor occupancy in a dose-dependent manner. Chlorpromazine administration resulted in the highest occupancy at D_2_ receptors, followed by 5-HT_2A_, H_1_, and mACh receptors. Meanwhile, risperidone administration produced the highest occupancy at 5-HT_2A_ receptors, followed by D_2_, H_1_, and mACh receptors. The effective dose 70 (ED_70_) values, corresponding to doses at which D_2_ receptor occupancy reaches 70% of the therapeutic range (65–80%) [6,7,8,9,10,11], were estimated to be 4.5 mg/kg and 1.5 mg/kg for chlorpromazine and risperidone, respectively; these doses were used in subsequent experiments.

### 3.2. Time Course of Brain D_2_, 5-HT_2A_, H_1_, and mACh Receptor Occupancy After Oral Administration of Chlorpromazine, Risperidone, or Their Combination in Rats

**Figure 2:**
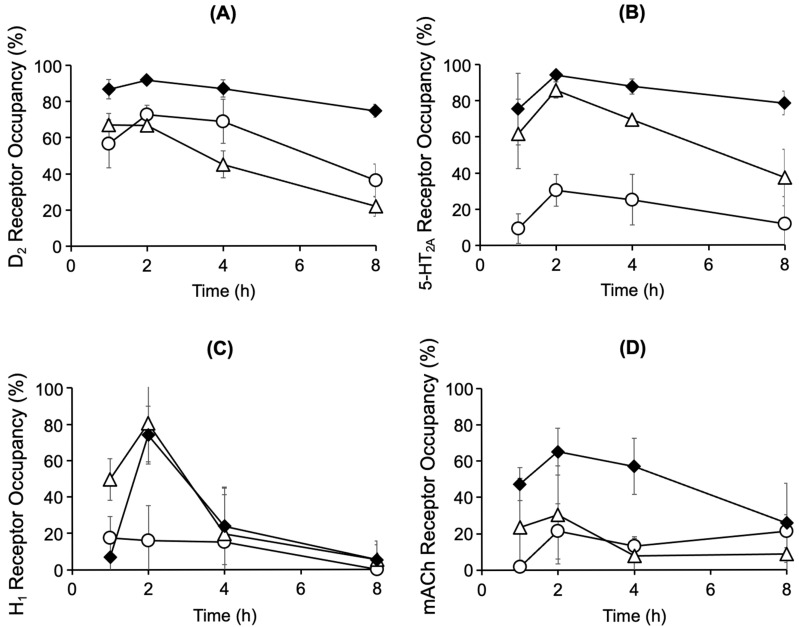
Time course of brain D_2_ (**A**), 5-HT_2A_ (**B**), H_1_ (**C**), and mACh (**D**) receptor occupancy after oral administration of chlorpromazine (4.5 mg/kg) (〇), risperidone (1.5 mg/kg) (△), or their combination (**◆**) in rats. The data are presented as the mean ± standard deviation from four rats at each point.

Figure 2A and Figure 2B show the time course of receptor occupancy in the rat brain after mono-administration of chlorpromazine (4.5 mg/kg, p.o.) or risperidone (1.5 mg/kg, p.o.), respectively. Striatal D_2_ receptor occupancy reached 73% and 67% 2 h after mono-administration of chlorpromazine or risperidone, respectively. After mono-administration of chlorpromazine, the occupancy of 5-HT_2A_, H_1_, and mACh receptors in the cerebral cortex remained below 30%. After mono-administration of risperidone, 5-HT_2A_ receptor occupancy reached its highest level of 86% and H_1_ receptor occupancy transiently increased to 80%. Compared with mono-administration, co-administration of chlorpromazine and risperidone increased the occupancy of the D_2_, 5-HT_2A_, and mACh receptors (Figure 2C). The D_2_ receptor occupancy exceeded 80% for up to 4 h after co-administration. Co-administration maintained a high 5-HT_2A_ receptor occupancy of 75–94%. mACh receptor occupancy, which was low after mono-administration, increased to 26–65% after co-administration.

### 3.3. Time Course of Brain Concentrations of Chlorpromazine, Risperidone, and Paliperidone After Oral Administration of Chlorpromazine, Risperidone, or Their Combination in Rats

**Figure 3:**
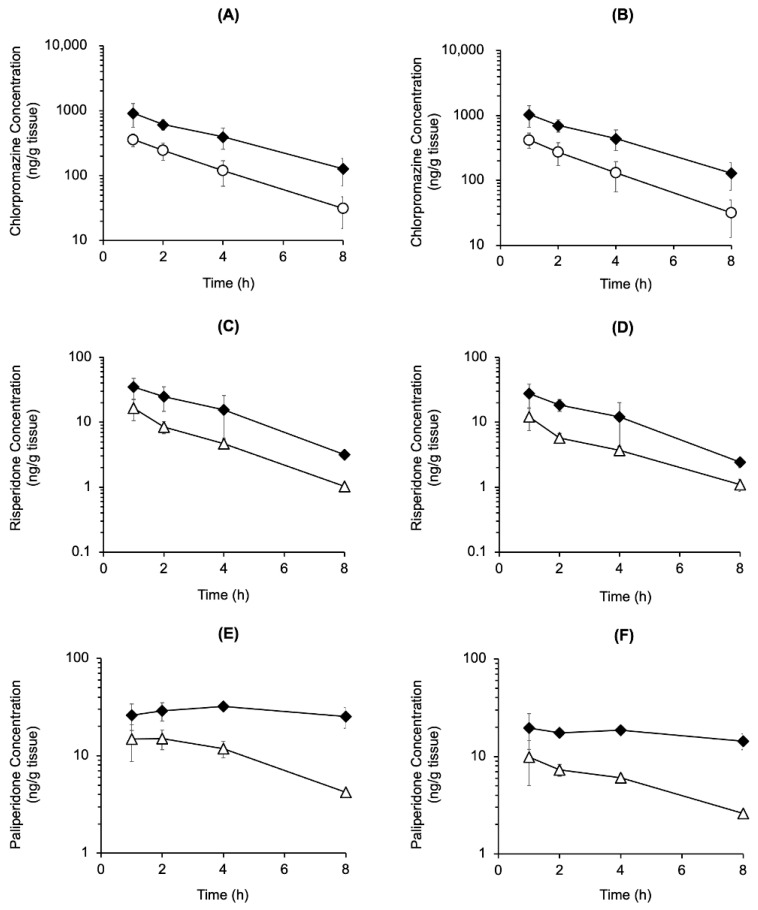
Time course of chlorpromazine, risperidone, and paliperidone concentrations in the cerebral cortex (**A**,**C**,**E**) and striatum (**B**,**D**,**F**) after the oral mono-administration of chlorpromazine (4.5 mg/kg) (〇), risperidone (1.5 mg/kg) (△), or their combination (**◆**) in rats. The data are presented as the mean ± standard deviation from four rats at each point.

The striatal and cerebral cortical concentrations of chlorpromazine, risperidone, and paliperidone, the active metabolite of risperidone, were measured after mono- and co-administration of chlorpromazine (4.5 mg/kg, p.o.) or risperidone (1.5 mg/kg, p.o.) (Figure 3). The concentrations were similar in both brain regions. Chlorpromazine concentrations in the striatum and cerebral cortex were two- to four-fold higher in rats co-administered risperidone than in those administered chlorpromazine alone (Figure 3A,B). Meanwhile, risperidone and paliperidone concentrations in the striatum and cerebral cortex were two- to six-fold higher in rats co-administered chlorpromazine than in those administered risperidone alone (Figure 3C–F).

### 3.4. Time Course of Plasma Concentrations of Chlorpromazine, Risperidone, and Paliperidone After Oral Administration of Chlorpromazine, Risperidone, or Their Combination in Rats

**Figure 4:**
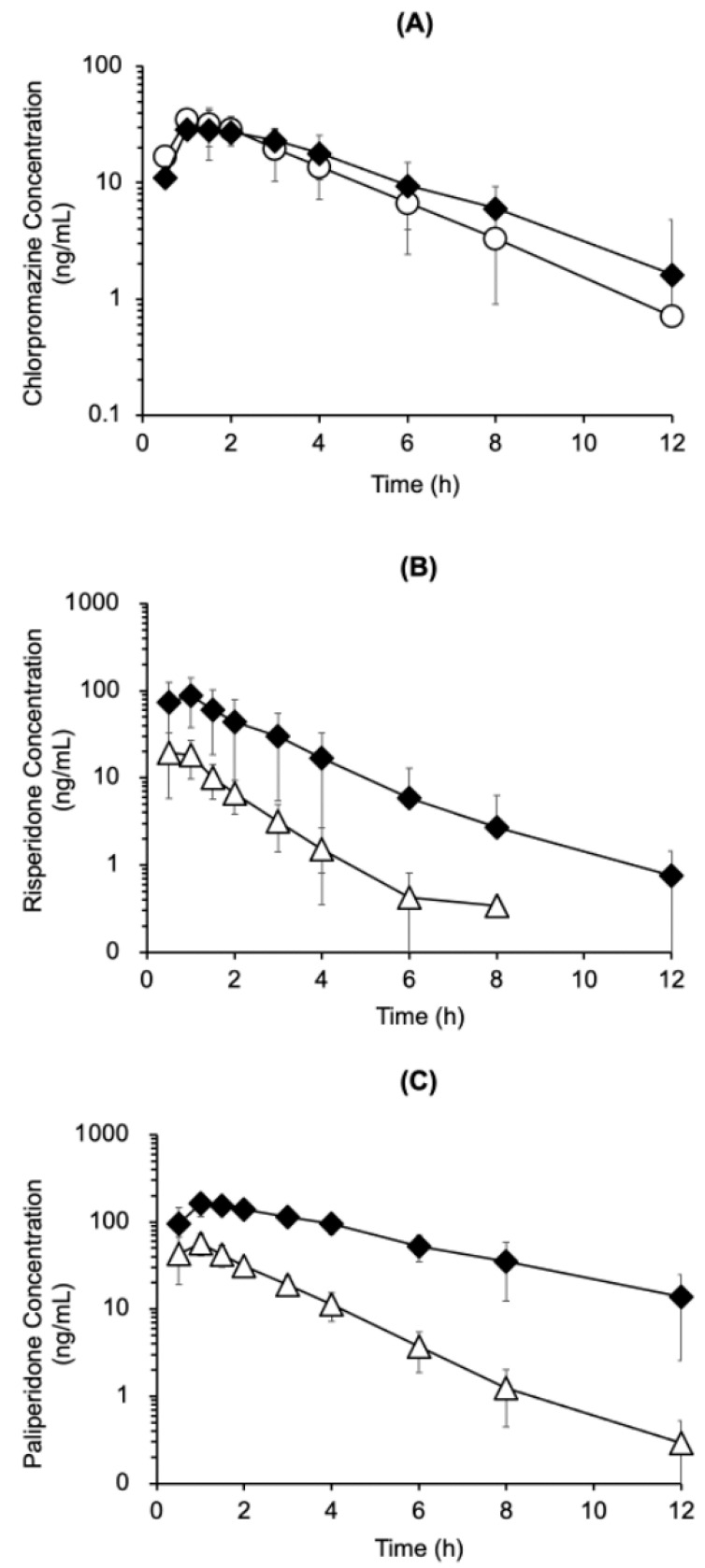
Time course of plasma chlorpromazine (**A**), risperidone (**B**), and paliperidone (**C**) concentrations after oral mono-administration of chlorpromazine (4.5 mg/kg) (〇), risperidone (1.5 mg/kg) (△), or their combination (**◆**) in rats. The data are presented as the mean ± standard deviation from five rats.

To evaluate the pharmacokinetic interaction between chlorpromazine and risperidone, the plasma concentrations of chlorpromazine, risperidone, and paliperidone were measured after the mono-administration of chlorpromazine (4.5 mg/kg, p.o.) or risperidone (1.5 mg/kg, p.o.) or their co-administration (Figure 4). Chlorpromazine concentrations in the plasma after co-administration with risperidone were comparable to those observed after mono-administration (Figure 4A). In contrast, risperidone and paliperidone concentrations in the plasma were higher after co-administration with chlorpromazine than after mono-administration, by up to 14-fold higher for risperidone (at 6 h) and up to 28-fold for paliperidone (at 8 h) (Figure 4B,C). Pharmacokinetic parameters were calculated using a non-compartmental analysis of the time courses of plasma chlorpromazine, risperidone, and paliperidone concentrations (Table 1). Compared with mono-administration of risperidone, the area under the curve from zero to infinity (AUC_0–∞_) of risperidone and paliperidone, maximum drug concentration (C_max_) of paliperidone after co-administration with chlorpromazine were significantly increased by 6.3- and 6.2-fold, and 2.9-fold, respectively, and the apparent oral clearance (CL/F) of risperidone was reduced by 5.9-fold. No significant differences in the pharmacokinetic parameters of chlorpromazine were observed between mono- and co-administration with risperidone. In the post-hoc power analysis comparing mono-administration of chlorpromazine, risperidone and their co-administration, the statistical power for chlorpromazine pharmacokinetic parameters (AUC_0–∞_, C_max_, T_max_, T_1/2_, and CL/F) was notably low, calculated as 0.14, 0.08, 0.19, 0.35, and 0.20, respectively. In contrast, the power values for risperidone were substantially higher: 0.83 for AUC_0–∞_, 0.88 for C_max_, 0.10 for T_max_, 0.80 for T_1/2_ and 0.98 for CL/F. Similarly, for paliperidone, the analysis demonstrated high statistical power for most parameters, with values of 0.99 for AUC_0–∞_, 0.99 for C_max_, 0.53 for T_max_ and 0.76 for T_1/2_.

## 4. Discussion

In this study, we investigated the pharmacodynamic and pharmacokinetic interactions between chlorpromazine and risperidone and found that the co-administration of chlorpromazine and risperidone increased the occupancy of multiple receptors in the brain and plasma concentrations of risperidone and its active metabolite, paliperidone, in rats.

To determine the doses that achieve the therapeutic range of striatal D_2_ receptor occupancy (65–80%) in schizophrenia drug therapy including chlorpromazine and risperidone in the human and rat study [6,7,8,9,10,11], we investigated the dose dependence of receptor occupancy. A dose-dependent increase in D_2_ receptor occupancy was observed after administration of chlorpromazine or raclopride (Figure 1). The ED_50_ values for D_2_ receptor occupancy following administration of chlorpromazine and risperidone were 2.5 mg/kg and 0.79 mg/kg, respectively. These values were comparable to previously reported ED_50_ values of 2.7–5.1 mg/kg for chlorpromazine and 0.1–0.4 mg/kg for risperidone, as determined using a radiolabeled or nonlabeled tracer [20]. The obtained ED_70_ values of chlorpromazine (4.5 mg/kg) and risperidone (1.5 mg/kg) were used as the doses for subsequent analysis. The order of receptor occupancy differed between chlorpromazine (D_2_ > 5-HT_2A_ > H_1_ > mACh receptors) and risperidone (5-HT_2A_ > D_2_ > H_1_ > mACh receptors), reflecting the distinct receptor-binding affinities of each drug [15,23].

The time course of striatal D_2_ receptor occupancy after administration of chlorpromazine (4.5 mg/kg) or risperidone (1.5 mg/kg) showed that chlorpromazine and risperidone reached the therapeutic range of D_2_ receptor occupancy (65–80%) at 2 h after administration (Figure 2). After chlorpromazine administration, 5-HT_2A_, H_1_, and mACh receptor occupancy remained low (<30%), whereas after risperidone administration, 5-HT_2A_ receptor occupancy exceeded D_2_ receptor occupancy, reflecting its higher binding affinity for 5-HT_2A_ receptors [15,23]. Compared with mono-administration, co-administration of chlorpromazine and risperidone increased D_2_ receptor occupancy, exceeding 80% at 4 h after administration, reaching a range of side effects that could cause extrapyramidal disorders. Co-administration increased 5-HT_2A_ receptor occupancy by 94% within 2–4 h. Although the side effects of excessive 5-HT_2A_ receptor blockade have not been clarified, studies have reported that such inhibition prolongs non-REM sleep, indicating possible centrally mediated side effects [24]. Although no increase in H_1_ receptor occupancy was observed after co-administration with chlorpromazine, risperidone caused a transient increase in H_1_ receptor occupancy to 80%, indicating a potential for transient sedation through H_1_ receptor blockade [16]. Interestingly, co-administration increased the concentrations of risperidone and its active metabolite paliperidone, yet H_1_ receptor occupancy remained largely unchanged, and the underlying mechanism remains unclear. In addition, co-administration increased mACh receptor occupancy to 60%. The elevated occupancy suggests a potential risk of central anticholinergic effects, such as cognitive impairment [17,18], which are unlikely to occur with mono-administration. However, as these findings are based on a single-dose rat study, their relevance to humans remains uncertain and should be interpreted with caution.

The concentrations of chlorpromazine, risperidone, and the active metabolite paliperidone in the cerebral cortex and striatum were elevated by co-administration compared to mono-administration (Figure 3), suggesting that in addition to pharmacodynamic interactions, pharmacokinetic interactions occurred between chlorpromazine and risperidone. The cerebral cortex/plasma concentration ratios for risperidone at 2 h were 1.9 ± 0.4 mL/g brain (mono-administration) and 1.1 ± 0.4 mL/g brain (co-administration), and the ratios for paliperidone were 0.4 ± 0.1 mL/g brain (mono-administration) and 0.3 ± 0.1 mL/g brain (co-administration) (mean ± SD, *n* = 4). To clarify the interaction in blood-brain barrier transport, it is necessary to verify changes in blood-brain barrier transport by measuring unbound drug concentrations. However, the lack of an increase in the brain-to-plasma concentration ratio suggests that the increase in brain concentrations of risperidone and paliperidone is more influenced by increased systemic exposure than by changes in blood-brain barrier transport.

For pharmacokinetic interaction analysis, the time courses of plasma drug concentrations was measured after mono- and co-administration. Co-administration with chlorpromazine significantly increased the plasma concentrations of risperidone and/or paliperidone (AUC_0–∞_ and C_max_) and decreased the oral clearance of risperidone (CL/F) (Figure 4 and Table 1). The AUC ratio of the metabolite to the parent compound (AUC_0–∞_ of paliperidone / AUC_0–∞_ of risperidone) was 4.04 after mono-administration and 4.00 after co-administration. On the other hand, human CYP2D6 inhibition inhibits the metabolism of risperidone to paliperidone [13]. These results may suggest species differences between rats and humans in the inhibition of the metabolism of risperidone to paliperidone. Although the molecular mechanisms of these pharmacokinetic interactions remain unclear, increased the AUC_0–∞_ and C_max_ of risperidone and/or paliperidone by chlorpromazine occurred accompanied by reduced oral clearance of risperidone in this rat study.

Overall, this rat study suggests that co-administration of chlorpromazine and risperidone may increase the risk of adverse reactions due to both pharmacodynamic interactions and pharmacokinetic interactions associated with elevated drug concentrations. While chlorpromazine-equivalent values are useful for assessing additive pharmacodynamic effects, they do not account for pharmacokinetic interactions and may therefore underestimate the overall impact. In addition to D_2_ and 5-HT_2A_ receptors, attention should also be given to the risk of side effects mediated by non-target receptors, as risperidone transiently increased H_1_ receptor occupancy and co-administration elevated mACh receptor occupancy in rats.

However, several limitations should be noted. First, pharmacokinetic interactions observed in rats cannot be directly extrapolated to humans due to species-specific differences in CYP isoforms and plasma protein binding. Second, receptor occupancy was used as a surrogate for pharmacodynamic interactions, but functional effects on the central nervous system were not directly evaluated. Third, the number of animals used was minimal—four rats per group per time point for receptor occupancy and brain concentration measurements, and five rats per group for plasma concentration measurements. Therefore, further studies are needed to clarify the pharmacokinetic and pharmacodynamic interactions between chlorpromazine and risperidone involving both target and non-target brain receptors in humans. Furthermore, we hope to develop methods for extrapolating the phenomena observed in rats to humans based on elucidating the mechanisms of drug-drug interactions and species differences. These findings also underscore the importance of considering both pharmacokinetic and pharmacodynamic interactions in combination therapy of antipsychotics. Future translational studies are warranted to validate these observations in clinical settings and to refine receptor occupancy-based strategies for optimizing antipsychotic combinations.

## 5. Conclusions

This study showed that the co-administration of chlorpromazine and risperidone increased the occupancy of D_2_, 5-HT_2A_, and mACh receptors in the rat brain and increased the plasma concentrations of risperidone and its metabolite, paliperidone, accompanied by reduced oral clearance of risperidone. These findings highlight the importance of considering the potential for increased adverse effects with antipsychotic combination therapy, due to both pharmacokinetic and pharmacodynamic interactions involving by target and non-target brain receptors.

## Figures and Tables

**Table 1:** Pharmacokinetic parameters of chlorpromazine, risperidone, and paliperidone calculated via non-compartmental analysis in rats.

	Chlorpromazine			Risperidone			Paliperidone		
	Mono-Administration	Co-Administration	Geometric Mean Ratio	Mono-Administration	Co-Administration	Geometric Mean Ratio	Mono-Administration	Co-Administration	Geometric Mean Ratio
AUC_0–∞_ (ng·h/mL)	128.8 ± 38.6	148.3 ± 39.7	1.2 (0.8–1.3)	34.3 ± 18.6	215.7 ± 145.0 *	6.0 (2.7–13)	138.8 ± 43.9	863.8 ± 224.5 **	6.3 (4.5–10.8)
C_max_ (ng/mL)	36.5 ± 10.7	33.7 ± 9.3	0.9 (0.7–1.0)	21.3 ± 11.9	98.7 ± 56.3	4.6 (2.3–8.3)	58.8 ± 17.9	173.1 ± 31.1 **	3.0 (2.4–3.7)
T_max_ (h)	1.1 ± 0.2	1.5 ± 0.9	1.2 (0.7–1.4)	0.8 ± 0.3	0.7 ± 0.3	0.9 (0.5–1.0)	0.9 ± 0.2	1.2 ± 0.3	1.4 (1.0–1.5)
T_1/2_ (h)	2.0 ± 0.2	2.2 ± 0.3	1.1 (0.9–1.2)	0.9 ± 0.2	1.4 ± 0.4	1.5 (0.1–1.7)	1.4 ± 0.4	2.8 ± 1.2	1.9 (1.1–2.3)
CL/F (L/h)	5.9 ± 1.6	4.9 ± 1.5	0.8 (0.6–1.9)	8.2 ± 3.9	1.4 ± 0.8 **	0.2 (0.1–0.2)			

AUC_0–∞_: area under the curve from zero to infinity; C_max_: maximum drug concentration; T_max_: time to maximum drug concentration; T_1/2_: half-life; CL/F: apparent oral clearance. The data are presented as the mean ± standard deviation from five rats. Geometric mean ratios are shown with 95% confidence intervals. * *p* < 0.05, ** *p* < 0.01 vs. mono-administration with Student’s *t*-test with post hoc Bonferroni-Holm correction.

## Data Availability

The original contributions presented in this study are included in the article material. Further inquiries can be directed to the corresponding author.

## Supplementary Materials

The following supporting information can be downloaded at: https://www.mdpi.com/article/10.3390/biomedicines14010118/s1, The Supplementary Materials provide the analytical conditions and validation data for the methods employed in this study.

## Abbreviations

The following abbreviations are used in this manuscript:

D_2_	dopamine 2 receptors
5-HT_2A_	serotonin 2A receptor
H_1_	histamine 1 receptor
mACh	muscarinic acetylcholine receptor
CYP2D6	cytochrome P450 2D6 enzyme
LC-MS/MS	liquid chromatography-tandem mass spectrometry
3-QNB	3-quinuclidinyl benzilate
BP	binding potential
SB	specific binding
ED_70_	effective dose 70
p.o.	Per os; orally
AUC_0–∞_	curve from zero to infinity
T_1/2_	half-life
C_max_	maximum drug concentration
CL/F	apparent oral clearance
T_max_	time to maximum drug concentration
