# Supplementary material for: Analysis of Pharmacokinetic and Pharmacodynamic Interactions Between Chlorpromazine and Risperidone via Simultaneous Measurement of Multiple Receptor Occupancy in the Rat Brain
Source: Biomedicines. 2026 Jan 6;14(1):118. doi: 10.3390/biomedicines14010118 (PMC12838218; doi:10.3390/biomedicines14010118)
Supplement: Supplementary file 1 [file biomedicines-14-00118-s001.zip › biomedicines-4038246-supplementary.pdf]

biomedicines

Supplementary Materials: Analysis Conditions and Analytical Method Validation

This appendix presents the analysis conditions for the analytical method used in this study and validation data. The validation was performed according to ICH Q2(R1) guidelines and includes specificity, linearity, precision, accuracy and lower limit of quantitation (LLOQ) studies.

Section S1. Chromatographic and MS-MS Conditions

Analyses were performed using a QTRAP 4500 system (AB Sciex, Tokyo, Japan) coupled with a Nexera XR system (Shimadzu Co., Kyoto, Japan) consisting of a solvent delivery unit (LC-20ADXR), degasser (DGU-20A3R), column oven (CTO-20AC), and autosampler (SIL-20ACXR). The analytical column used was Shim-pack XR-ODS (2.0 × 30 mm, 2.2 μm, Shimadzu GLC Ltd., Tokyo, Japan) with Guard column (Security Guard ULTRA, 2.1 mm, Phenomenex, California, USA), which was maintained at 50℃. An aliquot of 5 μl of each sample and calibration standard was loaded on the column. The mobile phase consisted of (A) 0.1% formic acid in water and (B) acetonitrile containing 0.1% formic acid. The mobile phase was delivered at a flow rate of 0.4 mL/min under gradient conditions. The gradient program was as follows: 5% solvent B from 0 to 0.2 min, increased linearly to 80% solvent B from 0.2 to 3.2 min, and maintained at 80% solvent B from 3.2 to 4.0 min. The mobile phase was then returned to the initial conditions (5% solvent B) at 4.01 min. The total run time was 4.5 min.

The spectrometric measurements were made in positive mode and operated using MRM mode. The optimal settings are given in Table S1 and Table S2.

Table S1. Optimal instrumental settings.

**Source/Gas parameter**	
TEM (temperature)	500 ℃
GS1 (gas source 1)	30 psi
GS2 (gas source 2)	80 psi
CUR (curtain gas)	50 psi
CAD (collision gas)	8 psi
IS (ion spray voltage)	5,500 V
IHE (interface heater)	on

Table S2. Optimal compound related settings.

**Drugs**	**Q1 (*m*/*z*)**	**Q3 (*m*/*z*)**	**DP (v)**	**EP (v)**	**CE (v)**	**CXP (v)**
Raclopride	346.99	112.00	1	10	33	10
MDL-100907	374.13	356.20	26	10	27	10
Pyrilamine	286.15	121.00	6	10	29	6
3-QNB	338.09	128.00	76	10	51	10
Chlorpromazine	318.97	58.10	1	10	57	10
Risperidone	411.07	191.10	31	10	37	12
Paliperidone	427.03	207.00	26	10	37	14
Propranolol (IS)	260.08	116.00	56	10	25	10

Section S2. Analytical Method Validation

S2.1. Specificity

Specificity was evaluated by analyzing blank samples and samples spiked with potential interfering compounds to ensure that the analytical method specifically measures the target analyte without interference.

The method demonstrated excellent specificity with no interference from blank or potential impurities (Figure S1 and S2).

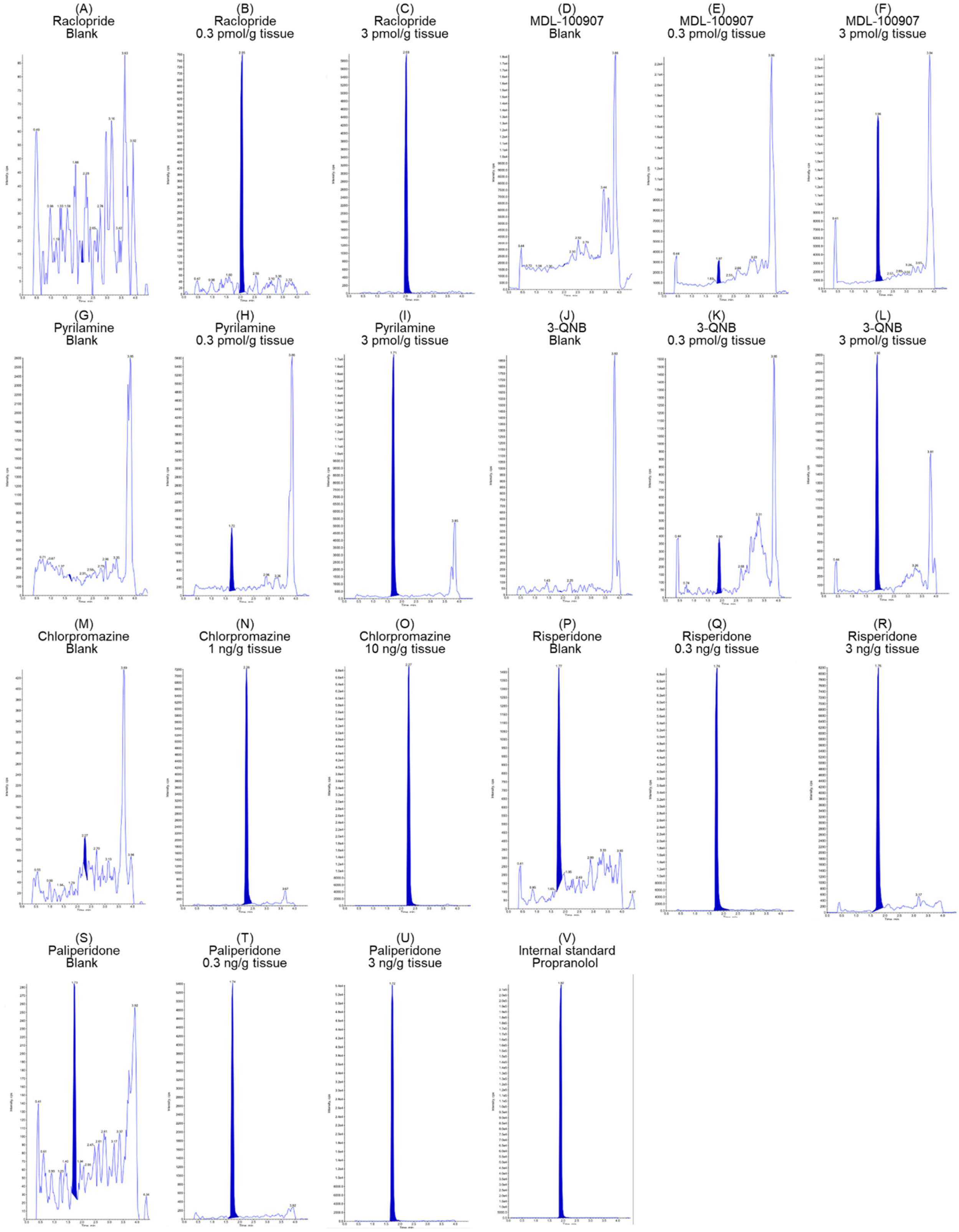

Figure S1. Representative chromatograms of tracers of brain samples spiked with tracers. Blank brain samples (Panel A, D, G, J, M, P, S), brain samples spiked with raclopride (Panel B, C), MDL-100907 (Panel E, F), pyrilamine (Panel H,

I), 3-QNB (Panel K, L) at concentration of 0.3 and 3 pmol/g tissue, chlorpromazine (Panel N, O) at concentration of 1 and 10 ng/g tissue, risperidone (Panel Q, R) and paliperidone (Panel T, U) at concentration of 0.3 and 3 ng/g tissue and propranolol (IS) (Panel V).

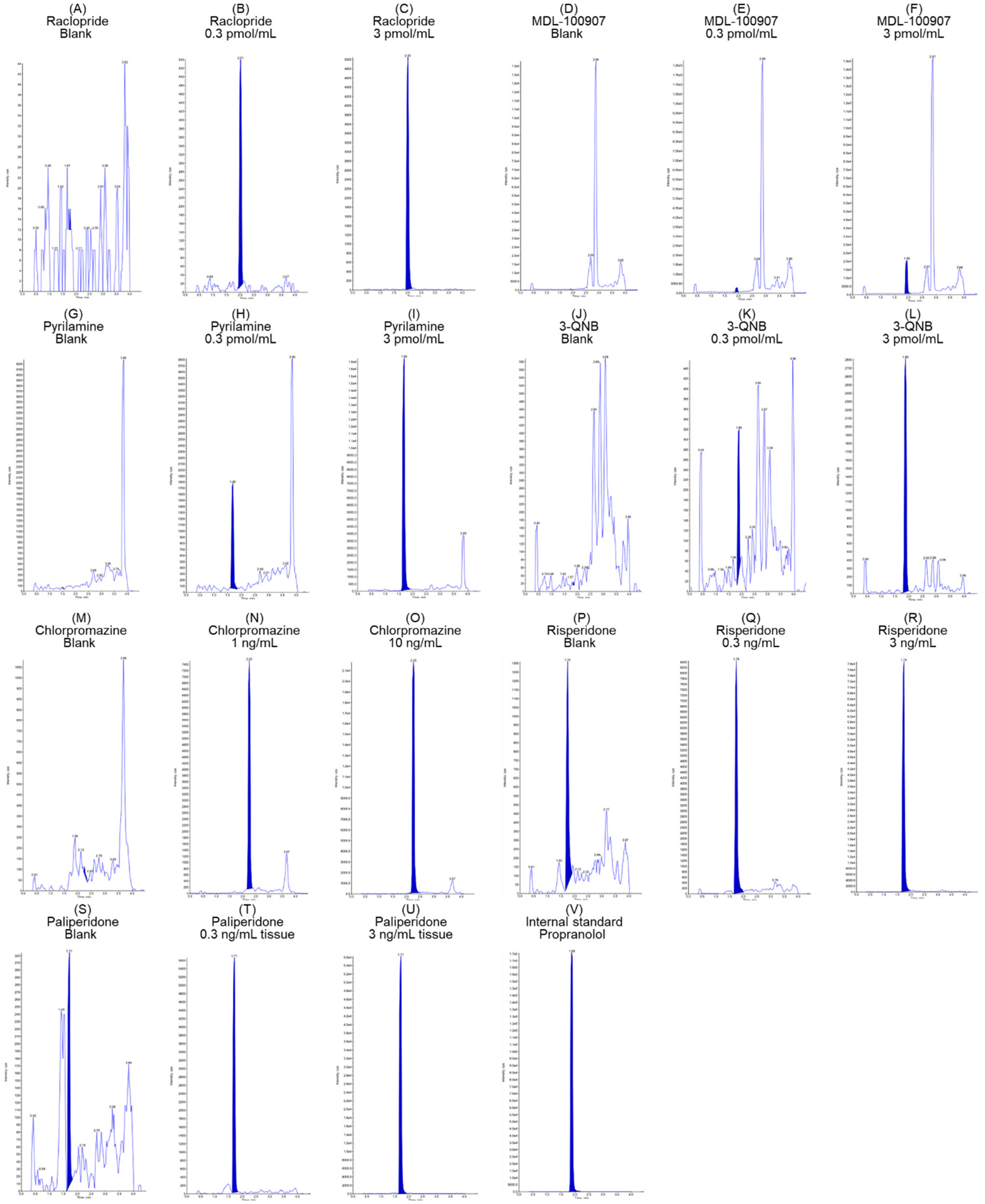

Figure S2. Representative chromatograms of plasma samples. Blank plasma samples (Panel A, D, G, J, M, P, S), plasma samples spiked with raclopride (Panel B, C), MDL-100907 (Panel E, F), pyrilamine (Panel H, I), 3-QNB (Panel

K, L) at concentration of 0.3 and 3 pmol/mL, chlorpromazine (Panel N, O) at concentration of 1 and 10 ng/mL, risperidone (Panel Q, R) and paliperidone (Panel T, U) at concentration of 0.3 and 3 ng/mL and propranolol (IS) (Panel V).

S2.2. Linearity

Calibration curves were generated using 5 different concentrations of each drug within the calibration range, and the data were analyzed by weighted linear regression (weighting factor, 1/x). LLOQ defined by the lowest concentration within the linear range of the calibration curves that gives an acceptable accuracy from 80% to 120% and a precision of < 20%. The method demonstrated good linearity over the range with $r^2 = 0.99$ (Table S3, S4).

Table S3. Calibration curves, linearity, and sensitivity of the assay of brain.

Drugs	Retention time (min)	Calibration range (pmol/g tissue or ng/g tissue)	Coefficient of determinations (R^2)	LLOQ (pmol/g)
Raclopride	2.03 ± 0.007	0.3–30	0.999 ± 0.001	0.3
MDL-100907	1.97 ± 0.007	0.3–30	0.992 ± 0.006	0.3
Pyrilamine	1.71 ± 0.007	0.3–300	0.994 ± 0.004	0.3
3-QNB	1.90 ± 0.007	0.3–300	0.994 ± 0.004	0.3
Chlorpromazine	2.28 ± 0.008	1-100	0.995 ± 0.005	1
Risperidone	1.76 ± 0.007	0.3–30	0.996 ± 0.003	0.3
Paliperidone	1.72 ± 0.008	0.3–30	0.998 ± 0.002	0.3
Propranolol (IS)	1.90 ± 0.005	—	—	—

The calibration curve units are pmol/g tissue for raclopride, MDL-100907, pyrilamine and 3-QNB, and ng/g tissue for chlorpromazine, risperidone and paliperidone.

Table S4. Calibration curves, linearity, and sensitivity of the assay of plasma.

Drugs	Retention time (min)	Calibration range (pmol/mL or ng/mL)	Coefficient of determinations (R^2)	LLOQ (ng/mL)
Raclopride	2.01 ± 0.005	0.3–30	0.994 ± 0.005	0.3
MDL-100907	1.95 ± 0.002	0.3–30	0.997 ± 0.001	0.3
Pyrilamine	1.68 ± 0.005	0.3–30	0.997 ± 0.004	0.3
3-QNB	1.89 ± 0.032	0.3–30	0.997 ± 0.001	0.3
Chlorpromazine	2.26 ± 0.009	1–100	0.996 ± 0.003	1
Risperidone	1.74 ± 0.002	0.3–30	0.995 ± 0.004	0.3
Paliperidone	1.71 ± 0.004	0.3–30	0.996 ± 0.004	0.3
Propranolol (IS)	1.88 ± 0.004	—	—	—

The calibration curve units are pmol/mL for raclopride, MDL-100907, pyrilamine and 3-QNB, and ng/mL for chlorpromazine, risperidone and paliperidone.

S2.3. Precision and Accuracy

The inter-day accuracy and precision of the assay were assessed by QC samples with 5 different determinations on 3 different days (Table S5 and S6).

Table S5. Precision and accuracy of the assay of brain.

Drugs	Concentration level (pmol/g tissue or ng/g tissue)	Precision (CV%)	Accuracy (relative bias%)
Raclopride	0.3	11.6	96.9 ± 11.3
	1	8.9	100 ± 9.0
	3	4.4	103 ± 4.6
	10	4.5	101 ± 4.5
	30	1.8	99.5 ± 1.8
MDL-100907	0.3	18.8	90.2 ± 16.7
	1	9.5	98.5 ± 9.4
	3	9.1	106 ± 9.6
	10	11.1	107 ± 11.8
	30	5.8	97.2 ± 5.5
Pyrilamine	0.3	8.7	83.4 ± 7.3
	1	7.9	97.7 ± 7.7
	3	8.6	107 ± 9.3
	10	8.3	108 ± 9.0
	30	7.9	101 ± 7.9
	100	9.0	105 ± 9.5
	300	6.8	95.1 ± 6.4
3-QNB	0.3	18.4	97.6 ± 18.0
	1	11.6	103 ± 12.0
	3	7.6	101 ± 7.7
	10	5.3	104 ± 5.5
	30	2.8	98.9 ± 2.7
	100	4.8	100 ± 4.8
	300	2.5	100 ± 2.6
Chlorpromazine	1	9.4	104 ± 9.8
	3	6.1	106 ± 6.6
	10	8.0	104 ± 8.3
	30	6.2	98.0 ± 6.0
	100	5.3	100 ± 5.3
Risperidone	0.3	13.8	86.9 ± 12.0
	1	7.8	102 ± 8.0
	3	6.1	103 ± 6.3
	10	6.9	104 ± 7.1
	30	5	98.6 ± 4.9
Paliperidone	0.3	10.6	99.1 ± 10.5
	1	5.2	104 ± 5.4
	3	8.1	106 ± 8.5
	10	3	98.0 ± 2.9
	30	8.2	96.0 ± 7.8

The concentration level units are pmol/g tissue for raclopride, MDL-100907, pyrilamine and 3-QNB, and ng/g tissue for chlorpromazine, risperidone and paliperidone.

Table S6. Precision and accuracy of the assay of plasma.

Drugs	Concentration level(pmol/mL or ng/mL)	Precision (CV%)	Accuracy (relative bias%)
Raclopride	0.3	14	89.2 ± 12.4
	1	7	104 ± 7.3
	3	6.3	106 ± 6.6
	10	9.5	102 ± 9.8
	30	5.8	98.6 ± 5.7
MDL-100907	0.3	10.1	97.8 ± 9.8
	1	5.5	97.0 ± 5.4
	3	5.6	105 ± 5.9
	10	8.1	100 ± 8.1
	30	3.3	99.4 ± 3.2
Pyrilamine	0.3	6.8	95.2 ± 6.5
	1	4.4	101 ± 4.5
	3	4.2	104 ± 4.3
	10	9.2	99.4 ± 9.1
	30	4.1	99.7 ± 4.0
	0.3	13.2	94.6 ± 12.3
	1	7.3	102 ± 7.4
3-QNB	3	6.6	103 ± 6.9
	10	3.3	99.6 ± 3.3
	30	3.4	99.9 ± 3.4
	1	4.7	99.4 ± 4.7
	3	9.4	107 ± 10.1
	10	8.1	102 ± 8.2
	30	7.6	101 ± 7.7
Chlorpromazine	100	6.5	96.5 ± 6.3
	0.3	7.9	95.9 ± 7.5
	1	12.2	107 ± 13.0
	3	7.5	104 ± 7.8
	10	11.2	103 ± 11.6
Risperidone	30	5.9	98.5 ± 5.8
	0.3	10.2	95.8 ± 9.7
	1	4.7	101 ± 4.8
	3	8.2	107 ± 8.9
	10	12	105 ± 12.6
Paliperidone	30	6.6	95.8 ± 6.3
	0.3	14	89.2 ± 12.4
	1	7	104 ± 7.3
	3	6.3	106 ± 6.6
	10	9.5	102 ± 9.8

The concentration level units are pmol/mL for raclopride, MDL-100907, pyrilamine and 3-QNB, and ng/mL for chlorpromazine, risperidone and paliperidone.
